# Supplementary material for: Combining Isotope Dilution and Standard Addition—Elemental Analysis in Complex Samples
Source: Molecules. 2021 Apr 30;26(9):2649. doi: 10.3390/molecules26092649 (PMC8124555; doi:10.3390/molecules26092649)
Supplement: Supplementary file 1 [file molecules-26-02649-s001.zip › Supplement_a_ID_ STDAdd.pdf]

Supplement:

Combining Isotope Dilution and Standard Addition – Elemental Analysis in Complex Samples

Christine Brauckmann[1], Axel Pramann[1,*], Olaf Rienitz[1], Alexander Schulze[1], Pranee Phukphatthanachai[2,3], and Jochen Vogl[2]

[1] Physikalisch-Technische Bundesanstalt (PTB), Bundesallee 100, 38116 Braunschweig, Germany
[2] Bundesanstalt für Materialforschung und -prüfung (BAM), Richard-Willstätter-Str.11, 12489 Berlin, Germany
[3] National Institute of Metrology (Thailand) (NIMT), 3/4-5 Moo 3, Klong 5, Klong Luang, Pathumthani 12120, Thailand
* Correspondence: axel.pramann@ptb.de; Tel.: +49-531-592-3219

(1) Derivation of equation (3)

This section presents the derivation of the combined isotope dilution and standard addition on a three-blend basis. The desired quantity is the mass fraction w_x of the sample x in a complex matrix. This derivation has been performed independently and from the scratch and yields finally the same equation as published earlier by *Pagliano* and *Meija* (*Metrologia* **2016**, *53*, 829; in this reference: eq. (9)). Thus, this derivation serves as a validation and conformation of the equation given by these authors with the advantage that the measurement of the isotope ratio in the spike material y becomes obsolete. A detailed discussion of the applicability of the final equation (3) is given in the main manuscript. The equation has been tested using the silicon/TMAH system using several sets of three different blends each (out of the five blends available). The calculation of the associated uncertainties when using the second, third, and fourth blend data triple were performed using the GUM Workbench Pro™ software (version 2.4.1 392, Metrodata GmbH, Germany) and compared to the application of the new combined IDMS/Standard addition approach (eqs. 1 and 2) presented in this paper.
The general idea is to develop an expression of the mass fraction w_x of the analyte element in the sample x (which might be present in a complex matrix). According to the standard addition principle, several blends b are prepared (b_1: blend 1) consisting of: the sample x, the reference solution z (with the same element as the sample which has the same or only slightly different isotopic composition as x), and the spike solution y (same element with preferably inverted isotope ratio). The isotope ratio of the monitor isotope (index 2) and the reference isotope (index 1) is given in equation (S.1). Here, $n_{b1,2}$ expresses the amount-of-substance of the isotope 2 (monitor) in the blend b1 and so forth. In our notation, the reference isotope 1 is that with the highest natural abundance. $n_{x1,2}$ expresses the amount-of-substance of the isotope 2 (monitor) in the sample x in blend b1; $n_{z1,2}$ is the amount-of-substance of the isotope 2 (monitor) in the reference in blend b1, and so forth. The respective $n_{xi,j}$ can be expressed by the respective amount-of-substance fractions x, masses m, mass fractions w, and molar masses M (eq. S.2).

$$R_{b1} = \frac{n_{b1,2}}{n_{b1,1}} = \frac{n_{x1,2} + n_{z1,2} + n_{y1,2}}{n_{x1,1} + n_{z1,1} + n_{y1,1}} \qquad \text{(S.1)}$$

$$n_{x1,2} = x_{x,2} \times \frac{m_{x1} w_x}{M_x} \qquad \text{(S.2)}$$

Inserting (S.2) into (S.1) yields

$$R_{\mathrm{b1}}=\frac{x_{\mathrm{x},2}\times\frac{m_{\mathrm{x1}}w_{\mathrm{x}}}{M_{\mathrm{x}}}+x_{\mathrm{z},2}\times\frac{m_{\mathrm{z1}}w_{\mathrm{z}}}{M_{\mathrm{z}}}+x_{\mathrm{y},2}\times\frac{m_{\mathrm{y1}}w_{\mathrm{y}}}{M_{\mathrm{y}}}}{x_{\mathrm{x},1}\times\frac{m_{\mathrm{x1}}w_{\mathrm{x}}}{M_{\mathrm{x}}}+x_{\mathrm{z},1}\times\frac{m_{\mathrm{z1}}w_{\mathrm{z}}}{M_{\mathrm{z}}}+x_{\mathrm{y},1}\times\frac{m_{\mathrm{y1}}w_{\mathrm{y}}}{M_{\mathrm{y}}}} \tag{S.3}$$

$x_{i,2}$ are expressed by $R_{i,2}\times x_{i,1}$:

$$R_{\mathrm{b1}}=\frac{R_{\mathrm{x},2}x_{\mathrm{x},1}\times\frac{m_{\mathrm{x1}}w_{\mathrm{x}}}{M_{\mathrm{x}}}+R_{\mathrm{z},2}x_{\mathrm{z},1}\times\frac{m_{\mathrm{z1}}w_{\mathrm{z}}}{M_{\mathrm{z}}}+R_{\mathrm{y},2}x_{\mathrm{y},1}\times\frac{m_{\mathrm{y1}}w_{\mathrm{y}}}{M_{\mathrm{y}}}}{x_{\mathrm{x},1}\times\frac{m_{\mathrm{x1}}w_{\mathrm{x}}}{M_{\mathrm{x}}}+x_{\mathrm{z},1}\times\frac{m_{\mathrm{z1}}w_{\mathrm{z}}}{M_{\mathrm{z}}}+x_{\mathrm{y},1}\times\frac{m_{\mathrm{y1}}w_{\mathrm{y}}}{M_{\mathrm{y}}}} \tag{S.4}$$

Rearranging and expanding gives

$$\begin{gathered}R_{\mathrm{b1}}\left(x_{\mathrm{x},1}\times\frac{m_{\mathrm{x1}}w_{\mathrm{x}}}{M_{\mathrm{x}}}+x_{\mathrm{z},1}\times\frac{m_{\mathrm{z1}}w_{\mathrm{z}}}{M_{\mathrm{z}}}+x_{\mathrm{y},1}\times\frac{m_{\mathrm{y1}}w_{\mathrm{y}}}{M_{\mathrm{y}}}\right)=\\ R_{\mathrm{b1}}x_{\mathrm{x},1}\times\frac{m_{\mathrm{x1}}w_{\mathrm{x}}}{M_{\mathrm{x}}}+R_{\mathrm{b1}}x_{\mathrm{z},1}\times\frac{m_{\mathrm{z1}}w_{\mathrm{z}}}{M_{\mathrm{z}}}+R_{\mathrm{b1}}x_{\mathrm{y},1}\times\frac{m_{\mathrm{y1}}w_{\mathrm{y}}}{M_{\mathrm{y}}}=\\ R_{\mathrm{x},2}x_{\mathrm{x},1}\times\frac{m_{\mathrm{x1}}w_{\mathrm{x}}}{M_{\mathrm{x}}}+R_{\mathrm{z},2}x_{\mathrm{z},1}\times\frac{m_{\mathrm{z1}}w_{\mathrm{z}}}{M_{\mathrm{z}}}+R_{\mathrm{y},2}x_{\mathrm{y},1}\times\frac{m_{\mathrm{y1}}w_{\mathrm{y}}}{M_{\mathrm{y}}}\end{gathered} \tag{S.5}$$

$$x_{\mathrm{x},1}\times\frac{m_{\mathrm{x1}}w_{\mathrm{x}}}{M_{\mathrm{x}}}\left(R_{\mathrm{b1}}-R_{\mathrm{x},2}\right)=x_{\mathrm{z},1}\times\frac{m_{\mathrm{z1}}w_{\mathrm{z}}}{M_{\mathrm{z}}}\left(R_{\mathrm{z},2}-R_{\mathrm{b1}}\right)+x_{\mathrm{y},1}\times\frac{m_{\mathrm{y1}}w_{\mathrm{y}}}{M_{\mathrm{y}}}\left(R_{\mathrm{y},2}-R_{\mathrm{b1}}\right) \tag{S.6}$$

Now, a first expression for w_{x} is obtained:

$$w_{\mathrm{x}}=w_{\mathrm{z}}\frac{m_{\mathrm{z1}}\left(R_{\mathrm{z},2}-R_{\mathrm{b1}}\right)}{m_{\mathrm{x1}}\left(R_{\mathrm{b1}}-R_{\mathrm{x},2}\right)}\frac{x_{\mathrm{z},1}M_{\mathrm{x}}}{x_{\mathrm{x},1}M_{\mathrm{z}}}+w_{\mathrm{y}}\frac{m_{\mathrm{y1}}\left(R_{\mathrm{y},2}-R_{\mathrm{b1}}\right)}{m_{\mathrm{x1}}\left(R_{\mathrm{b1}}-R_{\mathrm{x},2}\right)}\frac{x_{\mathrm{y},1}M_{\mathrm{x}}}{x_{\mathrm{x},1}M_{\mathrm{y}}} \tag{S.7}$$

$$x_{\mathrm{x},i}=x_{\mathrm{z},i}\quad\text{for all } i \tag{S.8}$$

$$w_{\mathrm{x}}=-w_{\mathrm{z}}\frac{m_{\mathrm{z1}}}{m_{\mathrm{x1}}}+w_{\mathrm{y}}\frac{m_{\mathrm{y1}}\left(R_{\mathrm{y},2}-R_{\mathrm{b1}}\right)}{m_{\mathrm{x1}}\left(R_{\mathrm{b1}}-R_{\mathrm{x},2}\right)}\frac{x_{\mathrm{y},1}M_{\mathrm{x}}}{x_{\mathrm{x},1}M_{\mathrm{y}}} \tag{S.9}$$

Rearranging equation (S.5) and separating w_{y} yields

$$\begin{gathered}R_{\mathrm{y},2}x_{\mathrm{y},1}\times\frac{m_{\mathrm{y1}}w_{\mathrm{y}}}{M_{\mathrm{y}}}-R_{\mathrm{b1}}x_{\mathrm{y},1}\times\frac{m_{\mathrm{y1}}w_{\mathrm{y}}}{M_{\mathrm{y}}}=\\ R_{\mathrm{b1}}x_{\mathrm{x},1}\times\frac{m_{\mathrm{x1}}w_{\mathrm{x}}}{M_{\mathrm{x}}}-R_{\mathrm{x},2}x_{\mathrm{x},1}\times\frac{m_{\mathrm{x1}}w_{\mathrm{x}}}{M_{\mathrm{x}}}+R_{\mathrm{b1}}x_{\mathrm{z},1}\times\frac{m_{\mathrm{z1}}w_{\mathrm{z}}}{M_{\mathrm{z}}}-R_{\mathrm{z},2}x_{\mathrm{z},1}\times\frac{m_{\mathrm{z1}}w_{\mathrm{z}}}{M_{\mathrm{z}}}\end{gathered} \tag{S.10}$$

$$w_{\mathrm{y}} m_{\mathrm{y1}} \left(R_{\mathrm{y,2}} - R_{\mathrm{b1}} \right) \frac{x_{\mathrm{y,1}}}{M_{\mathrm{y}}} = w_{\mathrm{x}} m_{\mathrm{x1}} \left(R_{\mathrm{b1}} - R_{\mathrm{x,2}} \right) \frac{x_{\mathrm{x,1}}}{M_{\mathrm{x}}} + w_{\mathrm{z}} m_{\mathrm{z1}} \left(R_{\mathrm{b1}} - R_{\mathrm{z,2}} \right) \frac{x_{\mathrm{z,1}}}{M_{\mathrm{z}}} \tag{S.11}$$

$$w_{\mathrm{y}} = w_{\mathrm{x}} \frac{m_{\mathrm{x1}}}{m_{\mathrm{y1}}} \frac{\left(R_{\mathrm{b1}} - R_{\mathrm{x,2}} \right)}{\left(R_{\mathrm{y,2}} - R_{\mathrm{b1}} \right)} \frac{x_{\mathrm{x,1}} M_{\mathrm{y}}}{x_{\mathrm{y,1}} M_{\mathrm{x}}} + w_{\mathrm{z}} \frac{m_{\mathrm{z1}}}{m_{\mathrm{y1}}} \frac{\left(R_{\mathrm{b1}} - R_{\mathrm{z,2}} \right)}{\left(R_{\mathrm{y,2}} - R_{\mathrm{b1}} \right)} \frac{x_{\mathrm{z,1}} M_{\mathrm{y}}}{x_{\mathrm{y,1}} M_{\mathrm{z}}} \tag{S.12}$$

$$w_{\mathrm{y}} = w_{\mathrm{x}} \frac{m_{\mathrm{x2}}}{m_{\mathrm{y2}}} \frac{\left(R_{\mathrm{b2}} - R_{\mathrm{x,2}} \right)}{\left(R_{\mathrm{y,2}} - R_{\mathrm{b2}} \right)} \frac{x_{\mathrm{x,1}} M_{\mathrm{y}}}{x_{\mathrm{y,1}} M_{\mathrm{x}}} + w_{\mathrm{z}} \frac{m_{\mathrm{z2}}}{m_{\mathrm{y2}}} \frac{\left(R_{\mathrm{b2}} - R_{\mathrm{z,2}} \right)}{\left(R_{\mathrm{y,2}} - R_{\mathrm{b2}} \right)} \frac{x_{\mathrm{z,1}} M_{\mathrm{y}}}{x_{\mathrm{y,1}} M_{\mathrm{z}}} \tag{S.13}$$

Equating eq. (S.12) with (S.13) gives

$$\begin{aligned} & w_{\mathrm{x}} \frac{m_{\mathrm{x1}}}{m_{\mathrm{y1}}} \frac{\left(R_{\mathrm{b1}} - R_{\mathrm{x,2}} \right)}{\left(R_{\mathrm{y,2}} - R_{\mathrm{b1}} \right)} \frac{x_{\mathrm{x,1}} M_{\mathrm{y}}}{x_{\mathrm{y,1}} M_{\mathrm{x}}} + w_{\mathrm{z}} \frac{m_{\mathrm{z1}}}{m_{\mathrm{y1}}} \frac{\left(R_{\mathrm{b1}} - R_{\mathrm{z,2}} \right)}{\left(R_{\mathrm{y,2}} - R_{\mathrm{b1}} \right)} \frac{x_{\mathrm{z,1}} M_{\mathrm{y}}}{x_{\mathrm{y,1}} M_{\mathrm{z}}} = \\ & w_{\mathrm{x}} \frac{m_{\mathrm{x2}}}{m_{\mathrm{y2}}} \frac{\left(R_{\mathrm{b2}} - R_{\mathrm{x,2}} \right)}{\left(R_{\mathrm{y,2}} - R_{\mathrm{b2}} \right)} \frac{x_{\mathrm{x,1}} M_{\mathrm{y}}}{x_{\mathrm{y,1}} M_{\mathrm{x}}} + w_{\mathrm{z}} \frac{m_{\mathrm{z2}}}{m_{\mathrm{y2}}} \frac{\left(R_{\mathrm{b2}} - R_{\mathrm{z,2}} \right)}{\left(R_{\mathrm{y,2}} - R_{\mathrm{b2}} \right)} \frac{x_{\mathrm{z,1}} M_{\mathrm{y}}}{x_{\mathrm{y,1}} M_{\mathrm{z}}} \end{aligned} \tag{S.14}$$

$$w_{\mathrm{x}} \frac{m_{\mathrm{x1}}}{m_{\mathrm{y1}}} \frac{\left(R_{\mathrm{b1}} - R_{\mathrm{x,2}} \right)}{\left(R_{\mathrm{y,2}} - R_{\mathrm{b1}} \right)} \frac{x_{\mathrm{x,1}}}{M_{\mathrm{x}}} + w_{\mathrm{z}} \frac{m_{\mathrm{z1}}}{m_{\mathrm{y1}}} \frac{\left(R_{\mathrm{b1}} - R_{\mathrm{z,2}} \right)}{\left(R_{\mathrm{y,2}} - R_{\mathrm{b1}} \right)} \frac{x_{\mathrm{z,1}}}{M_{\mathrm{z}}} = w_{\mathrm{x}} \frac{m_{\mathrm{x2}}}{m_{\mathrm{y2}}} \frac{\left(R_{\mathrm{b2}} - R_{\mathrm{x,2}} \right)}{\left(R_{\mathrm{y,2}} - R_{\mathrm{b2}} \right)} \frac{x_{\mathrm{x,1}}}{M_{\mathrm{x}}} + w_{\mathrm{z}} \frac{m_{\mathrm{z2}}}{m_{\mathrm{y2}}} \frac{\left(R_{\mathrm{b2}} - R_{\mathrm{z,2}} \right)}{\left(R_{\mathrm{y,2}} - R_{\mathrm{b2}} \right)} \frac{x_{\mathrm{z,1}}}{M_{\mathrm{z}}} \tag{S.15}$$

$$w_{\mathrm{x}} \frac{m_{\mathrm{x1}}}{m_{\mathrm{y1}}} \frac{\left(R_{\mathrm{b1}} - R_{\mathrm{x,2}} \right)}{\left(R_{\mathrm{y,2}} - R_{\mathrm{b1}} \right)} \frac{x_{\mathrm{x,1}}}{M_{\mathrm{x}}} - w_{\mathrm{x}} \frac{m_{\mathrm{x2}}}{m_{\mathrm{y2}}} \frac{\left(R_{\mathrm{b2}} - R_{\mathrm{x,2}} \right)}{\left(R_{\mathrm{y,2}} - R_{\mathrm{b2}} \right)} \frac{x_{\mathrm{x,1}}}{M_{\mathrm{x}}} = w_{\mathrm{z}} \frac{m_{\mathrm{z2}}}{m_{\mathrm{y2}}} \frac{\left(R_{\mathrm{b2}} - R_{\mathrm{z,2}} \right)}{\left(R_{\mathrm{y,2}} - R_{\mathrm{b2}} \right)} \frac{x_{\mathrm{z,1}}}{M_{\mathrm{z}}} - w_{\mathrm{z}} \frac{m_{\mathrm{z1}}}{m_{\mathrm{y1}}} \frac{\left(R_{\mathrm{b1}} - R_{\mathrm{z,2}} \right)}{\left(R_{\mathrm{y,2}} - R_{\mathrm{b1}} \right)} \frac{x_{\mathrm{z,1}}}{M_{\mathrm{z}}} \tag{S.16}$$

$$w_{\mathrm{x}} \frac{x_{\mathrm{x,1}}}{M_{\mathrm{x}}} \left[\frac{m_{\mathrm{x1}}}{m_{\mathrm{y1}}} \frac{\left(R_{\mathrm{b1}} - R_{\mathrm{x,2}} \right)}{\left(R_{\mathrm{y,2}} - R_{\mathrm{b1}} \right)} - \frac{m_{\mathrm{x2}}}{m_{\mathrm{y2}}} \frac{\left(R_{\mathrm{b2}} - R_{\mathrm{x,2}} \right)}{\left(R_{\mathrm{y,2}} - R_{\mathrm{b2}} \right)} \right] = w_{\mathrm{z}} \frac{x_{\mathrm{z,1}}}{M_{\mathrm{z}}} \left[\frac{m_{\mathrm{z2}}}{m_{\mathrm{y2}}} \frac{\left(R_{\mathrm{b2}} - R_{\mathrm{z,2}} \right)}{\left(R_{\mathrm{y,2}} - R_{\mathrm{b2}} \right)} - \frac{m_{\mathrm{z1}}}{m_{\mathrm{y1}}} \frac{\left(R_{\mathrm{b1}} - R_{\mathrm{z,2}} \right)}{\left(R_{\mathrm{y,2}} - R_{\mathrm{b1}} \right)} \right] \tag{S.17}$$

$$\begin{aligned} & w_{\mathrm{x}} \frac{x_{\mathrm{x,1}}}{M_{\mathrm{x}}} \frac{m_{\mathrm{x1}} m_{\mathrm{y2}} \left(R_{\mathrm{b1}} - R_{\mathrm{x,2}} \right) \left(R_{\mathrm{y,2}} - R_{\mathrm{b2}} \right) - m_{\mathrm{x2}} m_{\mathrm{y1}} \left(R_{\mathrm{b2}} - R_{\mathrm{x,2}} \right) \left(R_{\mathrm{y,2}} - R_{\mathrm{b1}} \right)}{m_{\mathrm{y1}} m_{\mathrm{y2}} \left(R_{\mathrm{y,2}} - R_{\mathrm{b1}} \right) \left(R_{\mathrm{y,2}} - R_{\mathrm{b2}} \right)} = \\ & w_{\mathrm{z}} \frac{x_{\mathrm{z,1}}}{M_{\mathrm{z}}} \frac{m_{\mathrm{z2}} m_{\mathrm{y1}} \left(R_{\mathrm{b2}} - R_{\mathrm{z,2}} \right) \left(R_{\mathrm{y,2}} - R_{\mathrm{b1}} \right) - m_{\mathrm{z1}} m_{\mathrm{y2}} \left(R_{\mathrm{b1}} - R_{\mathrm{z,2}} \right) \left(R_{\mathrm{y,2}} - R_{\mathrm{b2}} \right)}{m_{\mathrm{y1}} m_{\mathrm{y2}} \left(R_{\mathrm{y,2}} - R_{\mathrm{b1}} \right) \left(R_{\mathrm{y,2}} - R_{\mathrm{b2}} \right)} \end{aligned} \tag{S.18}$$

Solving eq. (S.18) for w_{x} yields

$$w_{\mathrm{x}} = w_{\mathrm{z}} \frac{m_{\mathrm{z2}} m_{\mathrm{y1}} \left(R_{\mathrm{b2}} - R_{\mathrm{z,2}}\right)\left(R_{\mathrm{y,2}} - R_{\mathrm{b1}}\right) - m_{\mathrm{z1}} m_{\mathrm{y2}} \left(R_{\mathrm{b1}} - R_{\mathrm{z,2}}\right)\left(R_{\mathrm{y,2}} - R_{\mathrm{b2}}\right)}{m_{\mathrm{x1}} m_{\mathrm{y2}} \left(R_{\mathrm{b1}} - R_{\mathrm{x,2}}\right)\left(R_{\mathrm{y,2}} - R_{\mathrm{b2}}\right) - m_{\mathrm{x2}} m_{\mathrm{y1}} \left(R_{\mathrm{b2}} - R_{\mathrm{x,2}}\right)\left(R_{\mathrm{y,2}} - R_{\mathrm{b1}}\right)} \frac{x_{\mathrm{z,1}} M_{\mathrm{x}}}{x_{\mathrm{x,1}} M_{\mathrm{z}}} \tag{S.19}$$

Having a third blend b3, the following expression (eq. (S.20)) analogue to eq. (S11) is obtained

$$w_{\mathrm{y}} m_{\mathrm{y3}} \left(R_{\mathrm{y,2}} - R_{\mathrm{b3}}\right) \frac{x_{\mathrm{y,1}}}{M_{\mathrm{y}}} = w_{\mathrm{x}} m_{\mathrm{x3}} \left(R_{\mathrm{b3}} - R_{\mathrm{x,2}}\right) \frac{x_{\mathrm{x,1}}}{M_{\mathrm{x}}} + w_{\mathrm{z}} m_{\mathrm{z3}} \left(R_{\mathrm{b3}} - R_{\mathrm{z,2}}\right) \frac{x_{\mathrm{z,1}}}{M_{\mathrm{z}}} \tag{S.20}$$

$$w_{\mathrm{y}} m_{\mathrm{y3}} R_{\mathrm{y,2}} \frac{x_{\mathrm{y,1}}}{M_{\mathrm{y}}} - w_{\mathrm{y}} m_{\mathrm{y3}} R_{\mathrm{b3}} \frac{x_{\mathrm{y,1}}}{M_{\mathrm{y}}} = w_{\mathrm{x}} m_{\mathrm{x3}} \left(R_{\mathrm{b3}} - R_{\mathrm{x,2}}\right) \frac{x_{\mathrm{x,1}}}{M_{\mathrm{x}}} + w_{\mathrm{z}} m_{\mathrm{z3}} \left(R_{\mathrm{b3}} - R_{\mathrm{z,2}}\right) \frac{x_{\mathrm{z,1}}}{M_{\mathrm{z}}} \tag{S.21}$$

$$R_{\mathrm{y,2}} w_{\mathrm{y}} m_{\mathrm{y3}} \frac{x_{\mathrm{y,1}}}{M_{\mathrm{y}}} = w_{\mathrm{x}} m_{\mathrm{x3}} \left(R_{\mathrm{b3}} - R_{\mathrm{x,2}}\right) \frac{x_{\mathrm{x,1}}}{M_{\mathrm{x}}} + w_{\mathrm{z}} m_{\mathrm{z3}} \left(R_{\mathrm{b3}} - R_{\mathrm{z,2}}\right) \frac{x_{\mathrm{z,1}}}{M_{\mathrm{z}}} + w_{\mathrm{y}} m_{\mathrm{y3}} R_{\mathrm{b3}} \frac{x_{\mathrm{y,1}}}{M_{\mathrm{y}}} \tag{S.22}$$

$$R_{\mathrm{y,2}} = \frac{w_{\mathrm{x}}}{w_{\mathrm{y}}} \frac{m_{\mathrm{x3}}}{m_{\mathrm{y3}}} \left(R_{\mathrm{b3}} - R_{\mathrm{x,2}}\right) \frac{x_{\mathrm{x,1}} M_{\mathrm{y}}}{x_{\mathrm{y,1}} M_{\mathrm{x}}} + \frac{w_{\mathrm{z}}}{w_{\mathrm{y}}} \frac{m_{\mathrm{z3}}}{m_{\mathrm{y3}}} \left(R_{\mathrm{b3}} - R_{\mathrm{z,2}}\right) \frac{x_{\mathrm{z,1}} M_{\mathrm{y}}}{x_{\mathrm{y,1}} M_{\mathrm{z}}} + R_{\mathrm{b3}} \tag{S.23}$$

Rearranging eq. (S.11) yields

$$w_{\mathrm{y}} m_{\mathrm{y1}} R_{\mathrm{y,2}} \frac{x_{\mathrm{y,1}}}{M_{\mathrm{y}}} - w_{\mathrm{y}} m_{\mathrm{y1}} R_{\mathrm{b1}} \frac{x_{\mathrm{y,1}}}{M_{\mathrm{y}}} = w_{\mathrm{x}} m_{\mathrm{x1}} \left(R_{\mathrm{b1}} - R_{\mathrm{x,2}}\right) \frac{x_{\mathrm{x,1}}}{M_{\mathrm{x}}} + w_{\mathrm{z}} m_{\mathrm{z1}} \left(R_{\mathrm{b1}} - R_{\mathrm{z,2}}\right) \frac{x_{\mathrm{z,1}}}{M_{\mathrm{z}}} \tag{S.24}$$

$$R_{\mathrm{y,2}} = \frac{w_{\mathrm{x}}}{w_{\mathrm{y}}} \frac{m_{\mathrm{x1}}}{m_{\mathrm{y1}}} \left(R_{\mathrm{b1}} - R_{\mathrm{x,2}}\right) \frac{x_{\mathrm{x,1}} M_{\mathrm{y}}}{x_{\mathrm{y,1}} M_{\mathrm{x}}} + \frac{w_{\mathrm{z}}}{w_{\mathrm{y}}} \frac{m_{\mathrm{z1}}}{m_{\mathrm{y1}}} \left(R_{\mathrm{b1}} - R_{\mathrm{z,2}}\right) \frac{x_{\mathrm{z,1}} M_{\mathrm{y}}}{x_{\mathrm{y,1}} M_{\mathrm{z}}} + R_{\mathrm{b1}} \tag{S.25}$$

Equating eq. (S.25) with eq. (S.23) gives

$$\begin{aligned} &\frac{w_{\mathrm{x}}}{w_{\mathrm{y}}} \frac{m_{\mathrm{x3}}}{m_{\mathrm{y3}}} \left(R_{\mathrm{b3}} - R_{\mathrm{x,2}}\right) \frac{x_{\mathrm{x,1}} M_{\mathrm{y}}}{x_{\mathrm{y,1}} M_{\mathrm{x}}} + \frac{w_{\mathrm{z}}}{w_{\mathrm{y}}} \frac{m_{\mathrm{z3}}}{m_{\mathrm{y3}}} \left(R_{\mathrm{b3}} - R_{\mathrm{z,2}}\right) \frac{x_{\mathrm{z,1}} M_{\mathrm{y}}}{x_{\mathrm{y,1}} M_{\mathrm{z}}} + R_{\mathrm{b3}} \\ &= \frac{w_{\mathrm{x}}}{w_{\mathrm{y}}} \frac{m_{\mathrm{x1}}}{m_{\mathrm{y1}}} \left(R_{\mathrm{b1}} - R_{\mathrm{x,2}}\right) \frac{x_{\mathrm{x,1}} M_{\mathrm{y}}}{x_{\mathrm{y,1}} M_{\mathrm{x}}} + \frac{w_{\mathrm{z}}}{w_{\mathrm{y}}} \frac{m_{\mathrm{z1}}}{m_{\mathrm{y1}}} \left(R_{\mathrm{b1}} - R_{\mathrm{z,2}}\right) \frac{x_{\mathrm{z,1}} M_{\mathrm{y}}}{x_{\mathrm{y,1}} M_{\mathrm{z}}} + R_{\mathrm{b1}} \end{aligned} \tag{S.26}$$

$$\begin{aligned} &w_{\mathrm{y}} R_{\mathrm{b3}} - w_{\mathrm{y}} R_{\mathrm{b1}} = \\ &w_{\mathrm{x}} \frac{m_{\mathrm{x1}}}{m_{\mathrm{y1}}} \left(R_{\mathrm{b1}} - R_{\mathrm{x,2}}\right) \frac{x_{\mathrm{x,1}} M_{\mathrm{y}}}{x_{\mathrm{y,1}} M_{\mathrm{x}}} + w_{\mathrm{z}} \frac{m_{\mathrm{z1}}}{m_{\mathrm{y1}}} \left(R_{\mathrm{b1}} - R_{\mathrm{z,2}}\right) \frac{x_{\mathrm{z,1}} M_{\mathrm{y}}}{x_{\mathrm{y,1}} M_{\mathrm{z}}} \\ &- w_{\mathrm{x}} \frac{m_{\mathrm{x3}}}{m_{\mathrm{y3}}} \left(R_{\mathrm{b3}} - R_{\mathrm{x,2}}\right) \frac{x_{\mathrm{x,1}} M_{\mathrm{y}}}{x_{\mathrm{y,1}} M_{\mathrm{x}}} - w_{\mathrm{z}} \frac{m_{\mathrm{z3}}}{m_{\mathrm{y3}}} \left(R_{\mathrm{b3}} - R_{\mathrm{z,2}}\right) \frac{x_{\mathrm{z,1}} M_{\mathrm{y}}}{x_{\mathrm{y,1}} M_{\mathrm{z}}} \end{aligned} \tag{S.27}$$

Solving for w_{y} gives

$$w_{\mathrm{y}} = w_{\mathrm{x}} \frac{m_{\mathrm{x1}}}{m_{\mathrm{y1}}} \frac{\left(R_{\mathrm{b1}} - R_{\mathrm{x,2}}\right)}{\left(R_{\mathrm{b3}} - R_{\mathrm{b1}}\right)} \frac{x_{\mathrm{x,1}} M_{\mathrm{y}}}{x_{\mathrm{y,1}} M_{\mathrm{x}}} + w_{\mathrm{z}} \frac{m_{\mathrm{z1}}}{m_{\mathrm{y1}}} \frac{\left(R_{\mathrm{b1}} - R_{\mathrm{z,2}}\right)}{\left(R_{\mathrm{b3}} - R_{\mathrm{b1}}\right)} \frac{x_{\mathrm{z,1}} M_{\mathrm{y}}}{x_{\mathrm{y,1}} M_{\mathrm{z}}}$$
$$- w_{\mathrm{x}} \frac{m_{\mathrm{x3}}}{m_{\mathrm{y3}}} \frac{\left(R_{\mathrm{b3}} - R_{\mathrm{x,2}}\right)}{\left(R_{\mathrm{b3}} - R_{\mathrm{b1}}\right)} \frac{x_{\mathrm{x,1}} M_{\mathrm{y}}}{x_{\mathrm{y,1}} M_{\mathrm{x}}} - w_{\mathrm{z}} \frac{m_{\mathrm{z3}}}{m_{\mathrm{y3}}} \frac{\left(R_{\mathrm{b3}} - R_{\mathrm{z,2}}\right)}{\left(R_{\mathrm{b3}} - R_{\mathrm{b1}}\right)} \frac{x_{\mathrm{z,1}} M_{\mathrm{y}}}{x_{\mathrm{y,1}} M_{\mathrm{z}}} \quad \text{(S.28)}$$

The analogue expression for blend b2 is:

$$w_{\mathrm{y}} = w_{\mathrm{x}} \frac{m_{\mathrm{x2}}}{m_{\mathrm{y2}}} \frac{\left(R_{\mathrm{b2}} - R_{\mathrm{x,2}}\right)}{\left(R_{\mathrm{b3}} - R_{\mathrm{b2}}\right)} \frac{x_{\mathrm{x,1}} M_{\mathrm{y}}}{x_{\mathrm{y,1}} M_{\mathrm{x}}} + w_{\mathrm{z}} \frac{m_{\mathrm{z2}}}{m_{\mathrm{y2}}} \frac{\left(R_{\mathrm{b2}} - R_{\mathrm{z,2}}\right)}{\left(R_{\mathrm{b3}} - R_{\mathrm{b2}}\right)} \frac{x_{\mathrm{z,1}} M_{\mathrm{y}}}{x_{\mathrm{y,1}} M_{\mathrm{z}}}$$
$$- w_{\mathrm{x}} \frac{m_{\mathrm{x3}}}{m_{\mathrm{y3}}} \frac{\left(R_{\mathrm{b3}} - R_{\mathrm{x,2}}\right)}{\left(R_{\mathrm{b3}} - R_{\mathrm{b2}}\right)} \frac{x_{\mathrm{x,1}} M_{\mathrm{y}}}{x_{\mathrm{y,1}} M_{\mathrm{x}}} - w_{\mathrm{z}} \frac{m_{\mathrm{z3}}}{m_{\mathrm{y3}}} \frac{\left(R_{\mathrm{b3}} - R_{\mathrm{z,2}}\right)}{\left(R_{\mathrm{b3}} - R_{\mathrm{b2}}\right)} \frac{x_{\mathrm{z,1}} M_{\mathrm{y}}}{x_{\mathrm{y,1}} M_{\mathrm{z}}} \quad \text{(S.29)}$$

Equating eq. (S.28) with eq. (S.29) gives

$$w_{\mathrm{x}} \frac{m_{\mathrm{x1}}}{m_{\mathrm{y1}}} \frac{\left(R_{\mathrm{b1}} - R_{\mathrm{x,2}}\right)}{\left(R_{\mathrm{b3}} - R_{\mathrm{b1}}\right)} \frac{x_{\mathrm{x,1}} M_{\mathrm{y}}}{x_{\mathrm{y,1}} M_{\mathrm{x}}} - w_{\mathrm{x}} \frac{m_{\mathrm{x3}}}{m_{\mathrm{y3}}} \frac{\left(R_{\mathrm{b3}} - R_{\mathrm{x,2}}\right)}{\left(R_{\mathrm{b3}} - R_{\mathrm{b1}}\right)} \frac{x_{\mathrm{x,1}} M_{\mathrm{y}}}{x_{\mathrm{y,1}} M_{\mathrm{x}}}$$
$$- w_{\mathrm{x}} \frac{m_{\mathrm{x2}}}{m_{\mathrm{y2}}} \frac{\left(R_{\mathrm{b2}} - R_{\mathrm{x,2}}\right)}{\left(R_{\mathrm{b3}} - R_{\mathrm{b2}}\right)} \frac{x_{\mathrm{x,1}} M_{\mathrm{y}}}{x_{\mathrm{y,1}} M_{\mathrm{x}}} + w_{\mathrm{x}} \frac{m_{\mathrm{x3}}}{m_{\mathrm{y3}}} \frac{\left(R_{\mathrm{b3}} - R_{\mathrm{x,2}}\right)}{\left(R_{\mathrm{b3}} - R_{\mathrm{b2}}\right)} \frac{x_{\mathrm{x,1}} M_{\mathrm{y}}}{x_{\mathrm{y,1}} M_{\mathrm{x}}}$$
$$= w_{\mathrm{z}} \frac{m_{\mathrm{z2}}}{m_{\mathrm{y2}}} \frac{\left(R_{\mathrm{b2}} - R_{\mathrm{z,2}}\right)}{\left(R_{\mathrm{b3}} - R_{\mathrm{b2}}\right)} \frac{x_{\mathrm{z,1}} M_{\mathrm{y}}}{x_{\mathrm{y,1}} M_{\mathrm{z}}} - w_{\mathrm{z}} \frac{m_{\mathrm{z3}}}{m_{\mathrm{y3}}} \frac{\left(R_{\mathrm{b3}} - R_{\mathrm{z,2}}\right)}{\left(R_{\mathrm{b3}} - R_{\mathrm{b2}}\right)} \frac{x_{\mathrm{z,1}} M_{\mathrm{y}}}{x_{\mathrm{y,1}} M_{\mathrm{z}}}$$
$$- w_{\mathrm{z}} \frac{m_{\mathrm{z1}}}{m_{\mathrm{y1}}} \frac{\left(R_{\mathrm{b1}} - R_{\mathrm{z,2}}\right)}{\left(R_{\mathrm{b3}} - R_{\mathrm{b1}}\right)} \frac{x_{\mathrm{z,1}} M_{\mathrm{y}}}{x_{\mathrm{y,1}} M_{\mathrm{z}}} + w_{\mathrm{z}} \frac{m_{\mathrm{z3}}}{m_{\mathrm{y3}}} \frac{\left(R_{\mathrm{b3}} - R_{\mathrm{z,2}}\right)}{\left(R_{\mathrm{b3}} - R_{\mathrm{b1}}\right)} \frac{x_{\mathrm{z,1}} M_{\mathrm{y}}}{x_{\mathrm{y,1}} M_{\mathrm{z}}} \quad \text{(S.30)}$$

$$w_{\mathrm{x}} \frac{m_{\mathrm{x1}}}{m_{\mathrm{y1}}} \frac{\left(R_{\mathrm{b1}} - R_{\mathrm{x,2}}\right)}{\left(R_{\mathrm{b3}} - R_{\mathrm{b1}}\right)} - w_{\mathrm{x}} \frac{m_{\mathrm{x3}}}{m_{\mathrm{y3}}} \frac{\left(R_{\mathrm{b3}} - R_{\mathrm{x,2}}\right)}{\left(R_{\mathrm{b3}} - R_{\mathrm{b1}}\right)} - w_{\mathrm{x}} \frac{m_{\mathrm{x2}}}{m_{\mathrm{y2}}} \frac{\left(R_{\mathrm{b2}} - R_{\mathrm{x,2}}\right)}{\left(R_{\mathrm{b3}} - R_{\mathrm{b2}}\right)} + w_{\mathrm{x}} \frac{m_{\mathrm{x3}}}{m_{\mathrm{y3}}} \frac{\left(R_{\mathrm{b3}} - R_{\mathrm{x,2}}\right)}{\left(R_{\mathrm{b3}} - R_{\mathrm{b2}}\right)}$$
$$= w_{\mathrm{z}} \frac{m_{\mathrm{z2}}}{m_{\mathrm{y2}}} \frac{\left(R_{\mathrm{b2}} - R_{\mathrm{z,2}}\right)}{\left(R_{\mathrm{b3}} - R_{\mathrm{b2}}\right)} \frac{x_{\mathrm{z,1}} M_{\mathrm{x}}}{x_{\mathrm{x,1}} M_{\mathrm{z}}} - w_{\mathrm{z}} \frac{m_{\mathrm{z3}}}{m_{\mathrm{y3}}} \frac{\left(R_{\mathrm{b3}} - R_{\mathrm{z,2}}\right)}{\left(R_{\mathrm{b3}} - R_{\mathrm{b2}}\right)} \frac{x_{\mathrm{z,1}} M_{\mathrm{x}}}{x_{\mathrm{x,1}} M_{\mathrm{z}}} - w_{\mathrm{z}} \frac{m_{\mathrm{z1}}}{m_{\mathrm{y1}}} \frac{\left(R_{\mathrm{b1}} - R_{\mathrm{z,2}}\right)}{\left(R_{\mathrm{b3}} - R_{\mathrm{b1}}\right)} \frac{x_{\mathrm{z,1}} M_{\mathrm{x}}}{x_{\mathrm{x,1}} M_{\mathrm{z}}} \quad \text{(S.31)}$$
$$+ w_{\mathrm{z}} \frac{m_{\mathrm{z3}}}{m_{\mathrm{y3}}} \frac{\left(R_{\mathrm{b3}} - R_{\mathrm{z,2}}\right)}{\left(R_{\mathrm{b3}} - R_{\mathrm{b1}}\right)} \frac{x_{\mathrm{z,1}} M_{\mathrm{x}}}{x_{\mathrm{x,1}} M_{\mathrm{z}}}$$

Performing several rearrangements yields

$$w_{\mathrm{x}} \left[\frac{m_{\mathrm{x1}}}{m_{\mathrm{y1}}} \frac{\left(R_{\mathrm{b1}} - R_{\mathrm{x,2}}\right)}{\left(R_{\mathrm{b3}} - R_{\mathrm{b1}}\right)} - \frac{m_{\mathrm{x3}}}{m_{\mathrm{y3}}} \frac{\left(R_{\mathrm{b3}} - R_{\mathrm{x,2}}\right)}{\left(R_{\mathrm{b3}} - R_{\mathrm{b1}}\right)} + \frac{m_{\mathrm{x3}}}{m_{\mathrm{y3}}} \frac{\left(R_{\mathrm{b3}} - R_{\mathrm{x,2}}\right)}{\left(R_{\mathrm{b3}} - R_{\mathrm{b2}}\right)} - \frac{m_{\mathrm{x2}}}{m_{\mathrm{y2}}} \frac{\left(R_{\mathrm{b2}} - R_{\mathrm{x,2}}\right)}{\left(R_{\mathrm{b3}} - R_{\mathrm{b2}}\right)} \right]$$
$$= w_{\mathrm{z}} \left[\frac{m_{\mathrm{z2}}}{m_{\mathrm{y2}}} \frac{\left(R_{\mathrm{b2}} - R_{\mathrm{z,2}}\right)}{\left(R_{\mathrm{b3}} - R_{\mathrm{b2}}\right)} - \frac{m_{\mathrm{z3}}}{m_{\mathrm{y3}}} \frac{\left(R_{\mathrm{b3}} - R_{\mathrm{z,2}}\right)}{\left(R_{\mathrm{b3}} - R_{\mathrm{b2}}\right)} + \frac{m_{\mathrm{z3}}}{m_{\mathrm{y3}}} \frac{\left(R_{\mathrm{b3}} - R_{\mathrm{z,2}}\right)}{\left(R_{\mathrm{b3}} - R_{\mathrm{b1}}\right)} - \frac{m_{\mathrm{z1}}}{m_{\mathrm{y1}}} \frac{\left(R_{\mathrm{b1}} - R_{\mathrm{z,2}}\right)}{\left(R_{\mathrm{b3}} - R_{\mathrm{b1}}\right)} \right] \frac{x_{\mathrm{z,1}} M_{\mathrm{x}}}{x_{\mathrm{x,1}} M_{\mathrm{z}}} \quad \text{(S.32)}$$

$$w_x \left[\frac{\begin{matrix} m_{x1}m_{y2}m_{y3}\left(R_{b1}-R_{x,2}\right)\left(R_{b3}-R_{b2}\right) \\ -m_{y1}m_{y2}m_{x3}\left(R_{b3}-R_{x,2}\right)\left(R_{b3}-R_{b2}\right) \\ +m_{y1}m_{y2}m_{x3}\left(R_{b3}-R_{x,2}\right)\left(R_{b3}-R_{b1}\right) \\ -m_{y1}m_{x2}m_{y3}\left(R_{b2}-R_{x,2}\right)\left(R_{b3}-R_{b1}\right) \end{matrix}}{m_{y1}m_{y2}m_{y3}\left(R_{b3}-R_{b1}\right)\left(R_{b3}-R_{b2}\right)} \right] = w_z \left[\frac{\begin{matrix} m_{y1}m_{z2}m_{y3}\left(R_{b3}-R_{b1}\right)\left(R_{b2}-R_{z,2}\right) \\ -m_{y1}m_{y2}m_{z3}\left(R_{b3}-R_{b1}\right)\left(R_{b3}-R_{z,2}\right) \\ +m_{y1}m_{y2}m_{z3}\left(R_{b3}-R_{z,2}\right)\left(R_{b3}-R_{b2}\right) \\ -m_{z1}m_{y2}m_{y3}\left(R_{b1}-R_{z,2}\right)\left(R_{b3}-R_{b2}\right) \end{matrix}}{m_{y1}m_{y2}m_{y3}\left(R_{b3}-R_{b1}\right)\left(R_{b3}-R_{b2}\right)} \right] \frac{x_{z,1}M_x}{x_{x,1}M_z}$$

(S.33)

Solving for w_x gives

$$w_x = w_z \left[\frac{\begin{matrix} m_{y1}m_{z2}m_{y3}\left(R_{b3}-R_{b1}\right)\left(R_{b2}-R_{z,2}\right) \\ -m_{y1}m_{y2}m_{z3}\left(R_{b3}-R_{b1}\right)\left(R_{b3}-R_{z,2}\right) \\ +m_{y1}m_{y2}m_{z3}\left(R_{b3}-R_{z,2}\right)\left(R_{b3}-R_{b2}\right) \\ -m_{z1}m_{y2}m_{y3}\left(R_{b1}-R_{z,2}\right)\left(R_{b3}-R_{b2}\right) \end{matrix}}{\begin{matrix} m_{x1}m_{y2}m_{y3}\left(R_{b1}-R_{x,2}\right)\left(R_{b3}-R_{b2}\right) \\ -m_{y1}m_{y2}m_{x3}\left(R_{b3}-R_{x,2}\right)\left(R_{b3}-R_{b2}\right) \\ +m_{y1}m_{y2}m_{x3}\left(R_{b3}-R_{x,2}\right)\left(R_{b3}-R_{b1}\right) \\ -m_{y1}m_{x2}m_{y3}\left(R_{b2}-R_{x,2}\right)\left(R_{b3}-R_{b1}\right) \end{matrix}} \right] \frac{x_{z,1}M_x}{x_{x,1}M_z}$$

(S.34)

$$w_x = w_z \left[\frac{\begin{matrix} m_{y1}m_{z2}m_{y3}\left(R_{b3}-R_{b1}\right)\left(R_{b2}-R_{z,2}\right)+m_{y1}m_{y2}m_{z3}\left[\left(R_{b3}-R_{z,2}\right)\left(R_{b3}-R_{b2}\right)-\left(R_{b3}-R_{b1}\right)\left(R_{b3}-R_{z,2}\right)\right]- \\ m_{z1}m_{y2}m_{y3}\left(R_{b1}-R_{z,2}\right)\left(R_{b3}-R_{b2}\right) \end{matrix}}{\begin{matrix} m_{x1}m_{y2}m_{y3}\left(R_{b1}-R_{x,2}\right)\left(R_{b3}-R_{b2}\right)+m_{y1}m_{y2}m_{x3}\left[\left(R_{b3}-R_{x,2}\right)\left(R_{b3}-R_{b1}\right)-\left(R_{b3}-R_{x,2}\right)\left(R_{b3}-R_{b2}\right)\right]- \\ m_{y1}m_{x2}m_{y3}\left(R_{b2}-R_{x,2}\right)\left(R_{b3}-R_{b1}\right) \end{matrix}} \right] \frac{x_{z,1}M_x}{x_{x,1}M_z}$$

(S.35)

$$w_x = w_z \left[\frac{\begin{matrix} m_{y1}m_{z2}m_{y3}\left(R_{b3}-R_{b1}\right)\left(R_{b2}-R_{z,2}\right)+m_{y1}m_{y2}m_{z3}\left(R_{b3}-R_{z,2}\right)\left[\left(R_{b3}-R_{b2}\right)-\left(R_{b3}-R_{b1}\right)\right]- \\ m_{z1}m_{y2}m_{y3}\left(R_{b1}-R_{z,2}\right)\left(R_{b3}-R_{b2}\right) \end{matrix}}{\begin{matrix} m_{x1}m_{y2}m_{y3}\left(R_{b1}-R_{x,2}\right)\left(R_{b3}-R_{b2}\right)+m_{y1}m_{y2}m_{x3}\left(R_{b3}-R_{x,2}\right)\left[\left(R_{b3}-R_{b1}\right)-\left(R_{b3}-R_{b2}\right)\right]- \\ m_{y1}m_{x2}m_{y3}\left(R_{b2}-R_{x,2}\right)\left(R_{b3}-R_{b1}\right) \end{matrix}} \right] \frac{x_{z,1}M_x}{x_{x,1}M_z}$$

(S.36)

$$w_x = w_z \left[\frac{m_{y1}m_{z2}m_{y3}\left(R_{b3}-R_{b1}\right)\left(R_{b2}-R_{z,2}\right)-m_{y1}m_{y2}m_{z3}\left(R_{b3}-R_{z,2}\right)\left(R_{b2}-R_{b1}\right)-m_{z1}m_{y2}m_{y3}\left(R_{b1}-R_{z,2}\right)\left(R_{b3}-R_{b2}\right)}{m_{x1}m_{y2}m_{y3}\left(R_{b1}-R_{x,2}\right)\left(R_{b3}-R_{b2}\right)-m_{y1}m_{y2}m_{x3}\left(R_{b3}-R_{x,2}\right)\left(R_{b1}-R_{b2}\right)-m_{y1}m_{x2}m_{y3}\left(R_{b2}-R_{x,2}\right)\left(R_{b3}-R_{b1}\right)} \right] \frac{x_{z,1}M_x}{x_{x,1}M_z}$$

(S.37)

$$w_x = w_z \left[\frac{m_{y1}m_{z2}m_{y3}\left(R_{b2}-R_{z,2}\right)\left(R_{b3}-R_{b1}\right)-m_{y1}m_{y2}m_{z3}\left(R_{b3}-R_{z,2}\right)\left(R_{b2}-R_{b1}\right)-m_{z1}m_{y2}m_{y3}\left(R_{b1}-R_{z,2}\right)\left(R_{b3}-R_{b2}\right)}{m_{x1}m_{y2}m_{y3}\left(R_{b1}-R_{x,2}\right)\left(R_{b3}-R_{b2}\right)+m_{y1}m_{y2}m_{x3}\left(R_{b3}-R_{x,2}\right)\left(R_{b2}-R_{b1}\right)-m_{y1}m_{x2}m_{y3}\left(R_{b2}-R_{x,2}\right)\left(R_{b3}-R_{b1}\right)} \right] \frac{x_{z,1}M_x}{x_{x,1}M_z}$$

(S.38)

Finally, eq. (3) results, which is exactly the same as eq. (9) in the paper of *Pagliano* and *Meija* (*Metrologia* **2016**, *53*, 829); however, a different notation was used and the sorting of the variables is done according to our requirements.

$$w_x = w_z \left[\frac{\begin{array}{l} m_{y1}m_{z2}m_{y3}\left(R_{b2}-R_{z,2}\right)\left(R_{b3}-R_{b1}\right) \\ -m_{y1}m_{y2}m_{z3}\left(R_{b3}-R_{z,2}\right)\left(R_{b2}-R_{b1}\right) \\ -m_{z1}m_{y2}m_{y3}\left(R_{b1}-R_{z,2}\right)\left(R_{b3}-R_{b2}\right) \end{array}}{\begin{array}{l} -m_{y1}m_{x2}m_{y3}\left(R_{b2}-R_{x,2}\right)\left(R_{b3}-R_{b1}\right) \\ +m_{y1}m_{y2}m_{x3}\left(R_{b3}-R_{x,2}\right)\left(R_{b2}-R_{b1}\right) \\ +m_{x1}m_{y2}m_{y3}\left(R_{b1}-R_{x,2}\right)\left(R_{b3}-R_{b2}\right) \end{array}} \right] \frac{x_{z,1}M_x}{x_{x,1}M_z} \qquad (3)$$

Table S.1: List of quantities and symbols in the general final equation (3)

**quantity**	**unit**	**description**
w_x	µg/g	mass fraction of the analyte in the sample x
w_z	µg/g	mass fraction of the analyte element in the reference solution z added to a blend b
m_{y1}	g	mass of spike solution y added to prepare blend b1
m_{z2}	g	mass of reference solution z added to prepare blend b2
m_{y3}	g	mass of spike solution y added to prepare blend b3
R_{b2}	mol/mol	isotope ratio (monitor/reference) in the blend b2
$R_{z,2}$	mol/mol	isotope ratio (monitor/reference) in the reference solution z
R_{b3}	mol/mol	isotope ratio (monitor/reference) in the blend b3

R_{b1}	mol/mol	isotope ratio (monitor/reference) in the blend b1
m_{y2}	g	mass of spike solution y added to prepare blend b2
m_{z3}	g	mass of reference solution z added to prepare blend b3
m_{z1}	g	mass of reference solution z added to prepare blend b1
m_{x2}	g	mass of sample x added to prepare blend b2
$R_{x,2}$	mol/mol	isotope ratio (monitor/reference) in the sample solution x
m_{x3}	g	mass of sample x added to prepare blend b3
m_{x1}	g	mass of sample x added to prepare blend b1
$x_{x,1}$	mol/mol	amount-of-substance fraction of isotope 1 (ref.) in the sample x
$x_{x,2}$	mol/mol	amount-of-substance fraction of isotope 2 (mon.) in the sample x
M_x	g/mol	molar mass of sample x
M_z	g/mol	molar mass of reference z (same element as sample)

(2) IDMS combined standard addition in the case of different isotopic patterns in sample and reference

This section presents the derivation of the combined isotope dilution and standard addition equation evaluated using a linear regression in case of different isotopic patterns in sample x and reference z. Basically it resembles the derivation in Appendix A of the main manuscript. The final equation (S.50) requires the additional knowledge of all isotope ratios in sample and reference.

$$R_{b,i} = \frac{n_{x2,i} + n_{z2,i} + n_{y2,i}}{n_{x1,i} + n_{z1,i} + n_{y1,i}} \tag{S.39}$$

$$R_{b,i} = \frac{x_{x2} n_{x,i} + x_{z2} n_{z,i} + x_{y2} n_{y,i}}{x_{x1} n_{x,i} + x_{z1} n_{z,i} + x_{y1} n_{y,i}} \tag{S.40}$$

$$R_{b,i} = \frac{x_{x2} \frac{m_{x,i} w_x}{M_x} + x_{z2} \frac{m_{z,i} w_z}{M_z} + x_{y2} \frac{m_{y,i} w_y}{M_y}}{x_{x1} \frac{m_{x,i} w_x}{M_x} + x_{z1} \frac{m_{z,i} w_z}{M_z} + x_{y1} \frac{m_{y,i} w_y}{M_y}} \tag{S.41}$$

$x_{x1} \neq x_{z1}, x_{x2} \neq x_{z2}, M_x \neq M_z$

$$R_{b,i}\left[\frac{x_{x1}w_x}{M_x}m_{x,i}+\frac{x_{z1}w_z}{M_z}m_{z,i}+\frac{x_{y1}w_y}{M_y}m_{y,i}\right]=\frac{x_{x2}w_x}{M_x}m_{x,i}+\frac{x_{z2}w_z}{M_z}m_{z,i}+\frac{x_{y2}w_y}{M_y}m_{y,i} \quad \text{(S.42)}$$

$$R_{b,i}\frac{1}{\sum R_x}\frac{w_x}{M_x}m_{x,i}+R_{b,i}\frac{1}{\sum R_z}\frac{w_z}{M_z}m_{z,i}+R_{b,i}\frac{1}{\sum R_y}\frac{w_y}{M_y}m_{y,i} \quad \text{(S.43)}$$

$$-\frac{R_x}{\sum R_x}\frac{w_x}{M_x}m_{x,i}-\frac{R_z}{\sum R_z}\frac{w_z}{M_z}m_{z,i}-\frac{R_y}{\sum R_y}\frac{w_y}{M_y}m_{y,i}=0 \quad \text{(S.44)}$$

$$m_{x,i}\frac{1}{\sum R_x}\frac{w_x}{M_x}\left(R_{b,i}-R_x\right)+m_{z,i}\frac{1}{\sum R_z}\frac{w_z}{M_z}\left(R_{b,i}-R_z\right)+m_{y,i}\frac{1}{\sum R_y}\frac{w_y}{M_y}\left(R_{b,i}-R_y\right)=0 \quad \text{(S.45)}$$

$$\frac{1}{\sum R_x}\frac{w_x}{M_x}+\frac{m_{z,i}}{m_{x,i}}\frac{1}{\sum R_z}\frac{w_z}{M_z}\frac{\left(R_{b,i}-R_z\right)}{\left(R_{b,i}-R_x\right)}+\frac{m_{y,i}}{m_{x,i}}\frac{1}{\sum R_y}\frac{w_y}{M_y}\frac{\left(R_{b,i}-R_y\right)}{\left(R_{b,i}-R_x\right)}=0 \quad \text{(S.46)}$$

$$\frac{m_{y,i}}{m_{x,i}}\frac{\left(R_y-R_{b,i}\right)}{\left(R_{b,i}-R_x\right)}=\frac{\sum R_y}{\sum R_x}\frac{w_x}{M_x}\frac{M_y}{w_y}+\frac{m_{z,i}}{m_{x,i}}\frac{\sum R_y}{\sum R_z}\frac{w_z}{M_z}\frac{M_y}{w_y}\frac{\left(R_{b,i}-R_z\right)}{\left(R_{b,i}-R_x\right)} \quad \text{(S.47)}$$

$$\underbrace{\frac{m_{y,i}}{m_{x,i}}\frac{\left(R_y-R_{b,i}\right)}{\left(R_{b,i}-R_x\right)}}_{=y_i}=\underbrace{\frac{m_{z,i}}{m_{x,i}}\frac{\left(R_{b,i}-R_z\right)}{\left(R_{b,i}-R_x\right)}}_{=x_i}\times\underbrace{\frac{\sum R_y}{\sum R_z}\frac{M_y}{M_z}\frac{w_z}{w_y}}_{=a_1}+\underbrace{\frac{\sum R_y}{\sum R_x}\frac{M_y}{M_x}\frac{w_x}{w_y}}_{=a_0} \quad \text{(S.48)}$$

$$\frac{a_0}{a_1}=\frac{\dfrac{\sum R_y}{\sum R_x}\dfrac{M_y}{M_x}\dfrac{w_x}{w_y}}{\dfrac{\sum R_y}{\sum R_z}\dfrac{M_y}{M_z}\dfrac{w_z}{w_y}}=\frac{w_x}{w_z}\frac{M_z\sum R_z}{M_x\sum R_x} \quad \text{(S.49)}$$

$$w_x=\frac{a_0}{a_1}\times w_z\times\frac{M_x\sum R_x}{M_z\sum R_z} \quad \text{(S.50)}$$

(3) Uncertainty $u(w_x)$ associated with the analyte mass fraction in the sample x according to equation (1) and (2)

This section shows how to calculate the uncertainty $u(w_x)$ associated with the analyte mass fraction in the sample according to the GUM [9] using equation (2) as the model equation.

$$w_x=\frac{a_0}{a_1}\times w_z \quad \text{(2)}$$

$$u(w_z, a_0) = 0 \quad \wedge \quad u(w_z, a_1) = 0 \tag{S.51}$$

$$u^2(w_x) = \left(\frac{\partial w_x}{\partial w_z}\right)^2 u^2(w_z) + \left(\frac{\partial w_x}{\partial a_0}\right)^2 u^2(a_0) + \left(\frac{\partial w_x}{\partial a_1}\right)^2 u^2(a_1) + 2\times\left(\frac{\partial w_x}{\partial a_0}\right)\left(\frac{\partial w_x}{\partial a_1}\right) u(a_0, a_1) \tag{S.52}$$

$$\frac{\partial w_x}{\partial w_z} = \frac{a_0}{a_1} = \frac{w_x}{w_z} \tag{S.53}$$

$$\frac{\partial w_x}{\partial a_0} = \frac{w_z}{a_1} = \frac{w_x}{a_0} \tag{S.54}$$

$$\frac{\partial w_x}{\partial a_1} = -\frac{a_0}{a_1^2} w_z = -\frac{w_x}{a_1} \tag{S.55}$$

$$u^2(w_x) = \left(\frac{w_x}{w_z}\right)^2 u^2(w_z) + \left(\frac{w_x}{a_0}\right)^2 u^2(a_0) + \left(-\frac{w_x}{a_1}\right)^2 u^2(a_1) + 2\times\left(\frac{w_x}{a_0}\right)\left(-\frac{w_x}{a_1}\right) u(a_0, a_1) \tag{S.56}$$

$$u^2(w_x) = w_x^2 \frac{u^2(w_z)}{w_z^2} + w_x^2 \left[\frac{1}{a_0^2} u^2(a_0) + \frac{1}{a_1^2} u^2(a_1) - 2\frac{1}{a_0 a_1} u(a_0, a_1)\right] \tag{S.57}$$

$$\frac{u^2(w_x)}{w_x^2} = \frac{u^2(w_z)}{w_z^2} + \left[\frac{1}{a_0^2} u^2(a_0) + \frac{1}{a_1^2} u^2(a_1) - 2\frac{1}{a_0 a_1} u(a_0, a_1)\right] \tag{S.58}$$

$$\frac{1}{a_1^2} = \frac{1}{a_0^2}\left(\frac{w_x}{w_z}\right)^2 \quad \wedge \quad \frac{1}{a_1} = \frac{1}{a_0}\left(\frac{w_x}{w_z}\right) \quad \wedge \quad \frac{1}{a_0 a_1} = \frac{1}{a_0^2}\left(\frac{w_x}{w_z}\right) \tag{S.59}$$

$$\frac{u^2(w_x)}{w_x^2} = \frac{u^2(w_z)}{w_z^2} + \frac{1}{a_0^2}\left[u^2(a_0) + \left(\frac{w_x}{w_z}\right)^2 u^2(a_1) - 2\left(\frac{w_x}{w_z}\right) u(a_0, a_1)\right] \tag{S.60}$$

When using a so-called ordinary least squares fit (OLS) the regression parameters a_0 and a_1 of the straight line are calculated from minimizing the residual standard deviation *S*:

$$y = a_0 + a_1 \times x \tag{S.61}$$

$$S^2 = \frac{\sum_{i=1}^{n}\left[y_i - (a_0 + a_1 \times x_i)\right]^2}{n-2} \tag{S.62}$$

$$\frac{\partial S^2}{\partial a_0} = 0 \quad \wedge \quad \frac{\partial S^2}{\partial a_1} = 0 \tag{S.63}$$

Solving the resulting system of linear equations for a_0 and a_1 yields:

$$a_0 = \frac{\sum_{i=1}^{n} y_i \cdot \sum_{i=1}^{n} x_i^2 - \sum_{i=1}^{n} x_i y_i \cdot \sum_{i=1}^{n} x_i}{n\sum_{i=1}^{n} x_i^2 - \left(\sum_{i=1}^{n} x_i\right)^2} \tag{S.64}$$

$$a_1 = \frac{n\sum_{i=1}^{n} x_i y_i - \sum_{i=1}^{n} x_i \cdot \sum_{i=1}^{n} y_i}{n\sum_{i=1}^{n} x_i^2 - \left(\sum_{i=1}^{n} x_i\right)^2} \tag{S.65}$$

The according variances and the covariance look like this:

$$u^2\left(a_0\right)=\frac{\sum_i x_i^2}{n\sum_i x_i^2-\left(\sum_i x_i\right)^2}S^2 \tag{S.66}$$

$$u^2\left(a_1\right)=\frac{n}{n\sum_i x_i^2-\left(\sum_i x_i\right)^2}S^2 \tag{S.67}$$

$$u\left(a_0,a_1\right)=-\frac{\sum_i x_i}{n\sum_i x_i^2-\left(\sum_i x_i\right)^2}S^2 \tag{S.68}$$

The following additional rearrangements (RA) help to simplify the above expressions:

RA1

$$\frac{\sum_{i=1}^n x_i^2}{n\sum_{i=1}^n x_i^2-\left(\sum_{i=1}^n x_i\right)^2}=\frac{\sum_{i=1}^n x_i^2-\frac{1}{n}\left(\sum_{i=1}^n x_i\right)^2+\frac{1}{n}\left(\sum_{i=1}^n x_i\right)^2}{n\times\left[\sum_{i=1}^n x_i^2-\frac{1}{n}\left(\sum_{i=1}^n x_i\right)^2\right]}$$

$$=\frac{\sum_{i=1}^n x_i^2-\frac{1}{n}\left(\sum_{i=1}^n x_i\right)^2+n\frac{\left(\sum_{i=1}^n x_i\right)^2}{n^2}}{n\times\left[\sum_{i=1}^n x_i^2-\frac{1}{n}\left(\sum_{i=1}^n x_i\right)^2\right]}=\frac{1}{n}+\frac{n\frac{\left(\sum_{i=1}^n x_i\right)^2}{n^2}}{n\times\left[\sum_{i=1}^n x_i^2-\frac{1}{n}\left(\sum_{i=1}^n x_i\right)^2\right]}$$

$$=\frac{1}{n}+\frac{n\overline{x}^2}{n\times\left[\sum_{i=1}^n x_i^2-\frac{1}{n}\left(\sum_{i=1}^n x_i\right)^2\right]}=\frac{1}{n}+\frac{\overline{x}^2}{\sum_{i=1}^n x_i^2-\frac{1}{n}\left(\sum_{i=1}^n x_i\right)^2}\quad\text{with}\quad \overline{x}=\frac{\sum_{i=1}^n x_i}{n}$$

RA2

$$\sum_{i=1}^{n} x_i^2 - \frac{1}{n}\left(\sum_{i=1}^{n} x_i\right)^2 = \sum_{i=1}^{n} x_i^2 - \frac{1}{n}\left(\sum_{i=1}^{n} x_i\right)^2 - \frac{1}{n}\left(\sum_{i=1}^{n} x_i\right)^2 + \frac{1}{n}\left(\sum_{i=1}^{n} x_i\right)^2$$

$$= \sum_{i=1}^{n} x_i^2 - 2\frac{1}{n}\left(\sum_{i=1}^{n} x_i\right)^2 + \frac{1}{n}\left(\sum_{i=1}^{n} x_i\right)^2 = \sum_{i=1}^{n} x_i^2 - 2\frac{\sum_{i=1}^{n} x_i}{n}\sum_{i=1}^{n} x_i + \frac{\left(\sum_{i=1}^{n} x_i\right)^2}{n^2} n$$

$$= \sum_{i=1}^{n} x_i^2 - 2\bar{x}\sum_{i=1}^{n} x_i + \bar{x}^2 n = \sum_{i=1}^{n} x_i^2 - 2\bar{x}\sum_{i=1}^{n} x_i + \bar{x}^2\sum_{i=1}^{n} 1 = \sum_{i=1}^{n} x_i^2 - \sum_{i=1}^{n} 2\bar{x}x_i + \sum_{i=1}^{n} \bar{x}^2$$

$$= \sum_{i=1}^{n}\left(x_i^2 - 2\bar{x}x_i + \bar{x}^2\right) = \sum_{i=1}^{n}\left(x_i - \bar{x}\right)^2$$

RA1 and RA2 applied to equations S.66 – S.68 yields:

$$u^2\left(a_0\right) = \frac{1}{n} + \frac{\bar{x}^2}{\sum_{i=1}^{n} x_i^2 - \frac{1}{n}\left(\sum_{i=1}^{n} x_i\right)^2} S^2 = \frac{1}{n} + \frac{\bar{x}^2}{\sum_{i=1}^{n}\left(x_i - \bar{x}\right)^2} S^2 \quad \text{(S.69)}$$

$$u^2\left(a_1\right) = \frac{n}{n\sum_{i=1}^{n}\left(x_i - \bar{x}\right)^2} S^2 = \frac{S^2}{\sum_{i=1}^{n}\left(x_i - \bar{x}\right)^2} \quad \text{(S.70)}$$

$$u\left(a_0, a_1\right) = -\frac{\sum_{i} x_i}{n\sum_{i=1}^{n}\left(x_i - \bar{x}\right)^2} S^2 = -\frac{\bar{x}\times S^2}{\sum_{i=1}^{n}\left(x_i - \bar{x}\right)^2} \quad \text{(S.71)}$$

Equations S.69 – S.71 in S.60 leads to:

$$\frac{u^2(w_{\mathrm{x}})}{w_{\mathrm{x}}^2} = \frac{u^2\left(w_{\mathrm{z}}\right)}{w_{\mathrm{z}}^2} + \frac{1}{a_0^2}\left[\frac{1}{n} + \frac{\bar{x}^2}{\sum_{i=1}^{n}\left(x_i - \bar{x}\right)^2} S^2 + \left(\frac{w_{\mathrm{x}}}{w_{\mathrm{z}}}\right)^2 \frac{S^2}{\sum_{i=1}^{n}\left(x_i - \bar{x}\right)^2} + 2\left(\frac{w_{\mathrm{x}}}{w_{\mathrm{z}}}\right)\frac{\bar{x}\times S^2}{\sum_{i=1}^{n}\left(x_i - \bar{x}\right)^2}\right] \quad \text{(S.72)}$$

$$\frac{u^2(w_{\mathrm{x}})}{w_{\mathrm{x}}^2} = \frac{u^2\left(w_{\mathrm{z}}\right)}{w_{\mathrm{z}}^2} + \frac{S^2}{a_0^2}\left[\frac{1}{n} + \frac{\bar{x}^2}{\sum_{i=1}^{n}\left(x_i - \bar{x}\right)^2} + \left(\frac{w_{\mathrm{x}}}{w_{\mathrm{z}}}\right)^2 \frac{1}{\sum_{i=1}^{n}\left(x_i - \bar{x}\right)^2} + 2\left(\frac{w_{\mathrm{x}}}{w_{\mathrm{z}}}\right)\frac{\bar{x}}{\sum_{i=1}^{n}\left(x_i - \bar{x}\right)^2}\right] \quad \text{(S.73)}$$

$$\frac{u^2(w_{\mathrm{x}})}{w_{\mathrm{x}}^2} = \frac{u^2\left(w_{\mathrm{z}}\right)}{w_{\mathrm{z}}^2} + \frac{S^2}{a_0^2}\left[\frac{1}{n} + \frac{\left(\frac{w_{\mathrm{x}}}{w_{\mathrm{z}}}\right)^2 + 2\left(\frac{w_{\mathrm{x}}}{w_{\mathrm{z}}}\right)\bar{x} + \bar{x}^2}{\sum_{i=1}^{n}\left(x_i - \bar{x}\right)^2}\right] \quad \text{(S.74)}$$

$$\left(\frac{u(w_{\mathrm{x}})}{w_{\mathrm{x}}}\right)^2 = \left(\frac{u\left(w_{\mathrm{z}}\right)}{w_{\mathrm{z}}}\right)^2 + \frac{S^2}{a_0^2}\left[\frac{1}{n} + \frac{\left(\frac{w_{\mathrm{x}}}{w_{\mathrm{z}}} + \bar{x}\right)^2}{\sum_{i=1}^{n}\left(x_i - \bar{x}\right)^2}\right] \quad \text{(S.75)}$$

Equation S.75 together with S.62 are necessary to calculate the uncertainty $u(w_X)$ associated with the mass fraction w_X of the analyte in the sample x. Since these equations are considerably bulky, an Excel® file (*.xlsm) is also provided as supplementary material. It contains a VBA® macro that makes the custom function MU_StdAdd_wz() available. This macro may be freely copied to any other files to facilitate a convenient uncertainty calculation for the combined IDMS standard addition method. The code looks as follows:

```
Function MU_StdAdd_wz(x_values As Range, y_values As Range, _
                    yIntercept As Double, slope As Double, _
                    wz As Double, urel_wz As Double) As Variant
'*********************************************************************************
'                               Function MU_StdAdd_wz
'*********************************************************************************
' Calculates the measurement uncertainty of the desired x-intercept of a
' straight line from a standard addition experiment

    Dim x(1 To 1000)           As Double
    Dim y(1 To 1000)           As Double
    Dim m                      As Integer
    Dim n                      As Integer
    Dim i                      As Integer
    Dim var                    As Variant
    Dim message                As Integer
    Dim Result                 As Variant
    Dim a0                     As Double
    Dim a1                     As Double
    Dim wX                     As Double
    Dim xMean
    Dim s                      As Double
    Dim D                      As Double

    '********************************read x-values
    m = 1
    For Each var In x_values
        x(m) = Val(var)
        m = m + 1
    Next var

    '********************************read y-values
    m = 1
    For Each var In y_values
        y(m) = Val(var)
        m = m + 1
    Next var

    n = m - 1                'number of data sets
```

```
    '********************************check number of data sets
    If n < 3 Then
        message = MsgBox("Please highlight at least 3 data sets!", _
                         vbInformation, "Information")
        Exit Function
    End If

    '********************************read parameters of straight line
    a0 = yIntercept   'a0
    a1 = slope        'a1

    '********************************calculate measurement uncertainty

    s = 0
    For i = 1 To n
        s = s + (y(i) - (a0 + a1 * x(i))) ^ 2
    Next i
    s = s / (n - 2)
    s = Sqr(s)

    xMean = 0
    For i = 1 To n
        xMean = xMean + x(i)
    Next i
    xMean = xMean / n

    D = 0
    For i = 1 To n
        D = D + (x(i) - xMean) ^ 2
    Next i

    wX = a0 / a1 * wz

    Result = Sqr(wX ^ 2 * (s ^ 2 / a0 ^ 2 * ((wX / wz + xMean) ^ 2 / D + 1 / n) _
                 + urel_wz ^ 2))

    '********************************Write result
    MU_StdAdd_wz = Result

'********************************************************************************
End Function
```

References

All references used in this supplement are listed in the main manuscript.
